# 2-[1-(*tert*-But­oxy­carbonyl)­pyrrolidin-2-yl]-4,4,5,5-tetra­methyl-4,5-dihydro-1*H*-imidazole-1-oxyl 3-oxide

**DOI:** 10.1107/S1600536810020672

**Published:** 2010-07-07

**Authors:** Ru Jiang, Hai-Bo Wang, Peng Gao, Lin-Lin Jing, Xiao-Li Sun

**Affiliations:** a Department of Chemistry, School of Pharmacy, Fourth Military Medical University, Changle West Road 17, 710032, Xi-An, People’s Republic of China

## Abstract

In the title compound, C_16_H_28_N_3_O_4_, the plane of the pyrrolidine ring system is twisted with respect to the plane of the nitronyl nitroxide unit, making a dihedral angle of 79.80 (6)°. The crystal structure is stabilized by C—H⋯O hydrogen bonds.

## Related literature

For the preparation of the title compound, see: Ullman *et al.* (1974 ▶). For the properties of nitronyl nitroxide radicals, see: Iqbal *et al.* (2009 ▶); Qin *et al.* (2009 ▶); Tanaka *et al.* (2007 ▶); Soule *et al.* (2007 ▶). For puckering parameters, see: Cremer & Pople (1975).
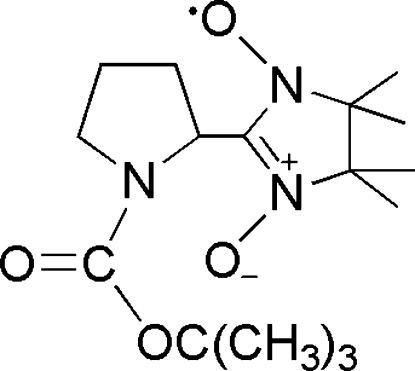


## Experimental

### 

#### Crystal data

C_16_H_28_N_3_O_4_
*M* *_r_* = 326.41
Monoclinic, 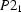

*a* = 6.1016 (12) Å
*b* = 10.392 (2) Å
*c* = 14.488 (3) Å
β = 101.312 (3)°
*V* = 900.8 (3) Å^3^
*Z* = 2
Mo *K*α radiation
μ = 0.09 mm^−1^
*T* = 296 K
0.36 × 0.28 × 0.17 mm

#### Data collection

Bruker SMART CCD area-detector diffractometer
4494 measured reflections
1686 independent reflections
1347 reflections with *I* > 2σ(*I*)
*R*_int_ = 0.048

#### Refinement

*R*[*F* ^2^ > 2σ(*F* ^2^)] = 0.042
*wR*(*F* ^2^) = 0.104
*S* = 0.97
1686 reflections
215 parameters
1 restraint
H-atom parameters constrained
Δρ_max_ = 0.18 e Å^−3^
Δρ_min_ = −0.20 e Å^−3^

### 

Data collection: *APEX2* (Bruker, 2007 ▶); cell refinement: *SAINT* (Bruker, 2007 ▶); data reduction: *SAINT*; program(s) used to solve structure: *SHELXS97* (Sheldrick, 2008 ▶); program(s) used to refine structure: *SHELXL97* (Sheldrick, 2008 ▶); molecular graphics: *ORTEPIII* (Burnett & Johnson, 1996 ▶) and *ORTEP-3 for Windows* (Farrugia, 1997 ▶); software used to prepare material for publication: *SHELXTL* (Sheldrick, 2008 ▶) and *PLATON* (Spek,2009 ▶).

## Supplementary Material

Crystal structure: contains datablocks I, global. DOI: 10.1107/S1600536810020672/dn2566sup1.cif

Structure factors: contains datablocks I. DOI: 10.1107/S1600536810020672/dn2566Isup2.hkl

Additional supplementary materials:  crystallographic information; 3D view; checkCIF report

## Figures and Tables

**Table 1:** Hydrogen-bond geometry (Å, °)

*D*—H⋯*A*	*D*—H	H⋯*A*	*D*⋯*A*	*D*—H⋯*A*
C2—H2*A*⋯O1	0.96	2.47	3.043 (5)	118
C3—H3*C*⋯O1	0.96	2.43	3.025 (4)	120
C16—H17*C*⋯O3^i^	0.96	2.48	3.390 (4)	157

Symmetry code: (i) 
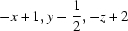
.

**Fig. 1.**
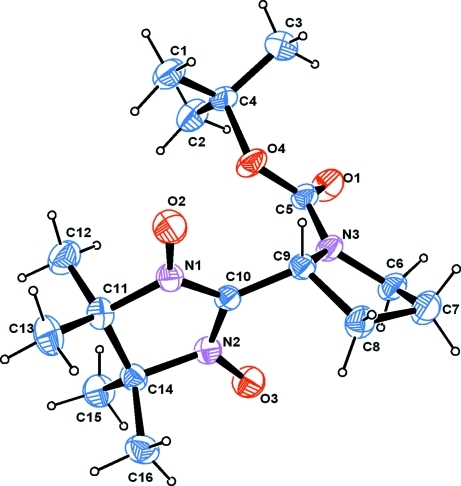
Molecular structure of the title compound (I), showing the atom labeling scheme. Displacement ellipsoids are drawn at the 30% probability level.

## supplementary crystallographic information

### Comment

Nitronyl nitroxide radical is a class of important functionalized molecule, which has characteristics of magnetism, anticancer, antiradiation and antioxidation, *etc *(Iqbal, *et al.*, 2009; Qin, *et al.*, 2009; Tanaka, *et al.*, 2007; Soule, *et al.*, 2007). The title compound has been be used for coordination with many metalcations, such as Mn^2+^, Cu^2+^ and Ni^2+ ^leading to form some molecule based magentic materials. The molecular structure of the title compound is shown in Fig1. The pyrrolidine ring and the nitronyl nitroxide ring are twisted with respect to each other making a dihedral angle of 79.80 (6)°. The crystal structure is stabilized by C—H···O hydrogen bonds (Table 1).

### Experimental

2,3-Dimethyl-2,3-bis(hydroxylamino) butane (1.48 g, 10.0 mmol) and *tert*-butyl-2-(hydroxymethyl) pyrrolidine-1-carboxylate (2.01 g, 10.0 mmol) were dissolved in methanol (Ullman, *et al.*, 1974). The reaction was stirred for 15 h at reflux temperature, then cooled to room temperature and filtered. The white powder was washed by methanol and suspended in a mixed solution of dichloromethane (30.0 ml) and water (30.0 ml). Then the reaction mixture was added to an aqueous solution of NaIO_4_ and stirred for 15 min in ice bath to give a blue solution. The aqueous phase was extracted with CH_2_Cl_2_ and the organic layer was combined and dried over MgSO_4_. Then the solvent was removed to give a dark red residue which was purified by a flash column chromatography with the elution of *n*-hexane/ ethyl acetate (1:3) to yield the title compound (I) as a dark blue powder. Single crystals of compound (I) were obtained from the mixed solution of *n*-heptane and dichloromethane (the ratio of volume is 1 to 1).

### Refinement

In both structures all the H atoms were discernible in the difference Fourier maps. However, they were constrained by riding model approximation. *C*—H_methyl_=0.96 Å; *C*—H_aryl_=0.93 Å; *U*_iso_H_methyl_ and *U*_iso _H_aryl _are 1.5 *U*_eq_ (C) and 1.2 *U*_eq_ (C), respectively.

### Crystal data

**Table:**

C_16_H_28_N_3_O_4_	*F*(000) = 354
*M**_r_* = 326.41	*D*_x_ = 1.203 Mg m^−^^3^
Monoclinic, *P*2_1_	Mo *K*α radiation, λ = 0.71073 Å
Hall symbol: P 2yb	Cell parameters from 1546 reflections
*a* = 6.1016 (12) Å	θ = 2.4–21.6°
*b* = 10.392 (2) Å	µ = 0.09 mm^−^^1^
*c* = 14.488 (3) Å	*T* = 296 K
β = 101.312 (3)°	Block, red
*V* = 900.8 (3) Å^3^	0.36 × 0.28 × 0.17 mm
*Z* = 2	

### Data collection

**Table:**

Bruker SMART CCD area-detector diffractometer	1347 reflections with *I* > 2σ(*I*)
Radiation source: fine-focus sealed tube	*R*_int_ = 0.048
graphite	θ_max_ = 25.1°, θ_min_ = 2.4°
phi and ω scans	*h* = −7→7
4494 measured reflections	*k* = −6→12
1686 independent reflections	*l* = −16→17

### Refinement

**Table:**

Refinement on *F*^2^	Secondary atom site location: difference Fourier map
Least-squares matrix: full	Hydrogen site location: inferred from neighbouring sites
*R*[*F*^2^ > 2σ(*F*^2^)] = 0.042	H-atom parameters constrained
*wR*(*F*^2^) = 0.104	*w* = 1/[σ^2^(*F*_o_^2^) + (0.0673*P*)^2^] where *P* = (*F*_o_^2^ + 2*F*_c_^2^)/3
*S* = 0.97	(Δ/σ)_max_ < 0.001
1686 reflections	Δρ_max_ = 0.18 e Å^−^^3^
215 parameters	Δρ_min_ = −0.19 e Å^−^^3^
1 restraint	Extinction correction: *SHELXL*, Fc^*^=kFc[1+0.001xFc^2^λ^3^/sin(2θ)]^-1/4^
Primary atom site location: structure-invariant direct methods	Extinction coefficient: 0.022 (5)

### Special details

**Table:**

Experimental. The absolute structure cannot be determined beacuse there are no atoms heaver than silicon in the molecular.
Geometry. All e.s.d.'s (except the e.s.d. in the dihedral angle between two l.s. planes) are estimated using the full covariance matrix. The cell e.s.d.'s are taken into account individually in the estimation of e.s.d.'s in distances, angles and torsion angles; correlations between e.s.d.'s in cell parameters are only used when they are defined by crystal symmetry. An approximate (isotropic) treatment of cell e.s.d.'s is used for estimating e.s.d.'s involving l.s. planes.
Refinement. Refinement of *F*^2^ against ALL reflections. The weighted *R*-factor *wR* and goodness of fit *S* are based on *F*^2^, conventional *R*-factors *R* are based on *F*, with *F* set to zero for negative *F*^2^. The threshold expression of *F*^2^ > σ(*F*^2^) is used only for calculating *R*-factors(gt) *etc*. and is not relevant to the choice of reflections for refinement. *R*-factors based on *F*^2^ are statistically about twice as large as those based on *F*, and *R*- factors based on ALL data will be even larger.

### Fractional atomic coordinates and isotropic or equivalent isotropic displacement parameters (Å^2^)

**Table:**

	*x*	*y*	*z*	*U*_iso_*/*U*_eq_	
N1	0.0929 (4)	0.3494 (3)	0.78508 (14)	0.0442 (6)	
N2	0.3330 (4)	0.4804 (2)	0.86782 (15)	0.0434 (6)	
N3	0.1712 (4)	0.6757 (2)	0.71424 (15)	0.0471 (6)	
O1	0.3991 (4)	0.7310 (3)	0.61493 (15)	0.0669 (7)	
O2	−0.0793 (3)	0.3080 (2)	0.72759 (14)	0.0615 (6)	
O3	0.4286 (4)	0.5857 (2)	0.90224 (15)	0.0621 (6)	
O4	0.2361 (4)	0.5320 (2)	0.61039 (13)	0.0575 (6)	
C1	0.2011 (6)	0.3558 (4)	0.5093 (2)	0.0656 (9)	
H1A	0.2562	0.3044	0.5640	0.098*	
H1B	0.2408	0.3160	0.4550	0.098*	
H1C	0.0414	0.3625	0.5004	0.098*	
C2	0.5524 (6)	0.4831 (4)	0.5354 (3)	0.0718 (10)	
H2A	0.6115	0.5689	0.5434	0.108*	
H2B	0.5934	0.4448	0.4809	0.108*	
H2C	0.6123	0.4327	0.5900	0.108*	
C3	0.1978 (7)	0.5764 (4)	0.4421 (2)	0.0799 (12)	
H3A	0.0387	0.5782	0.4375	0.120*	
H3B	0.2316	0.5450	0.3842	0.120*	
H3C	0.2569	0.6617	0.4541	0.120*	
C4	0.3028 (5)	0.4878 (3)	0.52248 (18)	0.0488 (8)	
C5	0.2803 (5)	0.6526 (3)	0.64280 (19)	0.0482 (7)	
C6	0.1631 (6)	0.8032 (3)	0.7549 (2)	0.0556 (8)	
H6A	0.1705	0.8699	0.7088	0.067*	
H6B	0.2842	0.8153	0.8086	0.067*	
C7	−0.0620 (7)	0.8035 (3)	0.7848 (3)	0.0690 (10)	
H9A	−0.0623	0.8630	0.8364	0.083*	
H9B	−0.1810	0.8269	0.7327	0.083*	
C8	−0.0877 (6)	0.6663 (3)	0.8153 (2)	0.0576 (9)	
H10A	−0.2443	0.6437	0.8084	0.069*	
H10B	−0.0150	0.6542	0.8806	0.069*	
C9	0.0256 (4)	0.5839 (3)	0.74985 (18)	0.0417 (7)	
H11	−0.0878	0.5518	0.6974	0.050*	
C10	0.1523 (4)	0.4731 (3)	0.79995 (17)	0.0385 (6)	
C11	0.2665 (5)	0.2596 (3)	0.8383 (2)	0.0506 (8)	
C12	0.4042 (6)	0.2131 (4)	0.7676 (2)	0.0705 (11)	
H15A	0.3088	0.1683	0.7172	0.106*	
H15B	0.5194	0.1560	0.7983	0.106*	
H15C	0.4709	0.2856	0.7427	0.106*	
C13	0.1549 (6)	0.1467 (4)	0.8763 (3)	0.0692 (10)	
H16A	0.0599	0.1775	0.9170	0.104*	
H16B	0.2669	0.0909	0.9110	0.104*	
H16C	0.0670	0.0999	0.8249	0.104*	
C14	0.3947 (4)	0.3532 (3)	0.91403 (18)	0.0450 (7)	
C15	0.6454 (5)	0.3379 (4)	0.9356 (2)	0.0635 (9)	
H18A	0.7020	0.3464	0.8786	0.095*	
H18B	0.6833	0.2544	0.9625	0.095*	
H18C	0.7105	0.4031	0.9795	0.095*	
C16	0.3053 (5)	0.3565 (4)	1.00551 (19)	0.0596 (9)	
H17A	0.1457	0.3658	0.9911	0.089*	
H17B	0.3702	0.4279	1.0432	0.089*	
H17C	0.3439	0.2778	1.0396	0.089*	

### Atomic displacement parameters (Å^2^)

**Table:**

	*U*^11^	*U*^22^	*U*^33^	*U*^12^	*U*^13^	*U*^23^
N1	0.0490 (13)	0.0398 (15)	0.0440 (12)	−0.0025 (12)	0.0091 (10)	0.0019 (11)
N2	0.0451 (13)	0.0403 (15)	0.0455 (12)	−0.0073 (13)	0.0104 (10)	0.0000 (12)
N3	0.0717 (15)	0.0350 (13)	0.0405 (11)	−0.0039 (13)	0.0252 (11)	−0.0009 (11)
O1	0.0936 (17)	0.0510 (14)	0.0679 (13)	−0.0193 (14)	0.0449 (12)	−0.0023 (12)
O2	0.0602 (12)	0.0519 (15)	0.0653 (12)	−0.0125 (11)	−0.0050 (10)	−0.0020 (11)
O3	0.0670 (13)	0.0529 (15)	0.0627 (12)	−0.0176 (13)	0.0034 (10)	−0.0058 (12)
O4	0.0907 (15)	0.0449 (13)	0.0470 (11)	−0.0064 (13)	0.0380 (10)	−0.0074 (10)
C1	0.087 (2)	0.058 (2)	0.0559 (17)	−0.002 (2)	0.0249 (15)	−0.0131 (17)
C2	0.072 (2)	0.069 (3)	0.078 (2)	0.002 (2)	0.0212 (16)	−0.020 (2)
C3	0.113 (3)	0.077 (3)	0.0487 (17)	0.017 (2)	0.0119 (17)	0.0065 (19)
C4	0.0631 (17)	0.052 (2)	0.0350 (13)	0.0042 (16)	0.0191 (11)	−0.0044 (14)
C5	0.0683 (19)	0.0390 (18)	0.0405 (13)	−0.0040 (16)	0.0183 (13)	−0.0013 (14)
C6	0.085 (2)	0.0382 (17)	0.0477 (15)	−0.0046 (18)	0.0227 (15)	−0.0050 (14)
C7	0.102 (3)	0.048 (2)	0.0658 (19)	0.009 (2)	0.0381 (18)	0.0000 (17)
C8	0.072 (2)	0.051 (2)	0.0579 (17)	0.0066 (18)	0.0331 (15)	0.0045 (16)
C9	0.0506 (15)	0.0379 (16)	0.0382 (12)	−0.0024 (14)	0.0123 (11)	0.0002 (13)
C10	0.0457 (14)	0.0363 (16)	0.0358 (12)	−0.0047 (14)	0.0141 (11)	0.0014 (13)
C11	0.0507 (17)	0.043 (2)	0.0583 (17)	0.0013 (15)	0.0113 (13)	0.0030 (14)
C12	0.073 (2)	0.069 (3)	0.072 (2)	0.013 (2)	0.0214 (17)	−0.014 (2)
C13	0.077 (2)	0.045 (2)	0.084 (2)	−0.0033 (19)	0.0152 (18)	0.0179 (19)
C14	0.0482 (15)	0.0446 (18)	0.0428 (14)	0.0008 (15)	0.0104 (11)	0.0058 (14)
C15	0.0514 (16)	0.071 (3)	0.0672 (18)	0.0057 (18)	0.0081 (14)	0.0002 (19)
C16	0.0642 (17)	0.071 (2)	0.0466 (15)	0.0057 (19)	0.0187 (13)	0.0097 (17)

### Geometric parameters (Å, °)

**Table:**

N1—O2	1.281 (3)	C6—H6B	0.9700
N1—C10	1.341 (4)	C7—C8	1.511 (5)
N1—C11	1.506 (4)	C7—H9A	0.9700
N2—O3	1.293 (3)	C7—H9B	0.9700
N2—C10	1.328 (3)	C8—C9	1.538 (4)
N2—C14	1.495 (4)	C8—H10A	0.9700
N3—C5	1.357 (4)	C8—H10B	0.9700
N3—C6	1.455 (4)	C9—C10	1.494 (4)
N3—C9	1.464 (4)	C9—H11	0.9800
O1—C5	1.211 (4)	C11—C13	1.513 (5)
O4—C5	1.347 (4)	C11—C12	1.526 (4)
O4—C4	1.484 (3)	C11—C14	1.557 (4)
C1—C4	1.502 (5)	C12—H15A	0.9600
C1—H1A	0.9600	C12—H15B	0.9600
C1—H1B	0.9600	C12—H15C	0.9600
C1—H1C	0.9600	C13—H16A	0.9600
C2—C4	1.499 (5)	C13—H16B	0.9600
C2—H2A	0.9600	C13—H16C	0.9600
C2—H2B	0.9600	C14—C15	1.509 (4)
C2—H2C	0.9600	C14—C16	1.529 (4)
C3—C4	1.523 (4)	C15—H18A	0.9600
C3—H3A	0.9600	C15—H18B	0.9600
C3—H3B	0.9600	C15—H18C	0.9600
C3—H3C	0.9600	C16—H17A	0.9600
C6—C7	1.518 (5)	C16—H17B	0.9600
C6—H6A	0.9700	C16—H17C	0.9600
			
O2—N1—C10	126.0 (2)	C7—C8—H10A	110.7
O2—N1—C11	122.0 (3)	C9—C8—H10A	110.7
C10—N1—C11	111.8 (2)	C7—C8—H10B	110.7
O3—N2—C10	125.4 (2)	C9—C8—H10B	110.7
O3—N2—C14	121.35 (19)	H10A—C8—H10B	108.8
C10—N2—C14	112.4 (2)	N3—C9—C10	112.4 (2)
C5—N3—C6	122.0 (3)	N3—C9—C8	103.4 (2)
C5—N3—C9	125.2 (2)	C10—C9—C8	112.4 (2)
C6—N3—C9	112.3 (2)	N3—C9—H11	109.5
C5—O4—C4	121.2 (2)	C10—C9—H11	109.5
C4—C1—H1A	109.5	C8—C9—H11	109.5
C4—C1—H1B	109.5	N2—C10—N1	109.4 (2)
H1A—C1—H1B	109.5	N2—C10—C9	126.2 (3)
C4—C1—H1C	109.5	N1—C10—C9	124.3 (2)
H1A—C1—H1C	109.5	N1—C11—C13	110.2 (2)
H1B—C1—H1C	109.5	N1—C11—C12	106.1 (2)
C4—C2—H2A	109.5	C13—C11—C12	110.2 (3)
C4—C2—H2B	109.5	N1—C11—C14	100.3 (2)
H2A—C2—H2B	109.5	C13—C11—C14	115.4 (3)
C4—C2—H2C	109.5	C12—C11—C14	113.9 (2)
H2A—C2—H2C	109.5	C11—C12—H15A	109.5
H2B—C2—H2C	109.5	C11—C12—H15B	109.5
C4—C3—H3A	109.5	H15A—C12—H15B	109.5
C4—C3—H3B	109.5	C11—C12—H15C	109.5
H3A—C3—H3B	109.5	H15A—C12—H15C	109.5
C4—C3—H3C	109.5	H15B—C12—H15C	109.5
H3A—C3—H3C	109.5	C11—C13—H16A	109.5
H3B—C3—H3C	109.5	C11—C13—H16B	109.5
O4—C4—C2	110.3 (2)	H16A—C13—H16B	109.5
O4—C4—C1	102.5 (2)	C11—C13—H16C	109.5
C2—C4—C1	111.7 (3)	H16A—C13—H16C	109.5
O4—C4—C3	108.9 (3)	H16B—C13—H16C	109.5
C2—C4—C3	112.3 (3)	N2—C14—C15	110.0 (3)
C1—C4—C3	110.7 (3)	N2—C14—C16	105.5 (3)
O1—C5—O4	127.0 (3)	C15—C14—C16	110.0 (2)
O1—C5—N3	123.4 (3)	N2—C14—C11	100.9 (2)
O4—C5—N3	109.6 (3)	C15—C14—C11	115.4 (3)
N3—C6—C7	102.8 (3)	C16—C14—C11	114.1 (2)
N3—C6—H6A	111.2	C14—C15—H18A	109.5
C7—C6—H6A	111.2	C14—C15—H18B	109.5
N3—C6—H6B	111.2	H18A—C15—H18B	109.5
C7—C6—H6B	111.2	C14—C15—H18C	109.5
H6A—C6—H6B	109.1	H18A—C15—H18C	109.5
C8—C7—C6	103.5 (3)	H18B—C15—H18C	109.5
C8—C7—H9A	111.1	C14—C16—H17A	109.5
C6—C7—H9A	111.1	C14—C16—H17B	109.5
C8—C7—H9B	111.1	H17A—C16—H17B	109.5
C6—C7—H9B	111.1	C14—C16—H17C	109.5
H9A—C7—H9B	109.0	H17A—C16—H17C	109.5
C7—C8—C9	105.0 (2)	H17B—C16—H17C	109.5
			
C5—O4—C4—C2	66.4 (4)	C11—N1—C10—C9	173.9 (2)
C5—O4—C4—C1	−174.5 (3)	N3—C9—C10—N2	50.4 (3)
C5—O4—C4—C3	−57.3 (4)	C8—C9—C10—N2	−65.8 (4)
C4—O4—C5—O1	−10.5 (5)	N3—C9—C10—N1	−132.5 (3)
C4—O4—C5—N3	169.9 (2)	C8—C9—C10—N1	111.3 (3)
C6—N3—C5—O1	8.7 (5)	O2—N1—C11—C13	−43.4 (4)
C9—N3—C5—O1	179.8 (3)	C10—N1—C11—C13	141.6 (3)
C6—N3—C5—O4	−171.7 (3)	O2—N1—C11—C12	75.8 (3)
C9—N3—C5—O4	−0.7 (4)	C10—N1—C11—C12	−99.2 (3)
C5—N3—C6—C7	148.7 (3)	O2—N1—C11—C14	−165.5 (2)
C9—N3—C6—C7	−23.4 (3)	C10—N1—C11—C14	19.5 (3)
N3—C6—C7—C8	34.8 (3)	O3—N2—C14—C15	−48.6 (3)
C6—C7—C8—C9	−34.1 (3)	C10—N2—C14—C15	141.3 (2)
C5—N3—C9—C10	69.1 (3)	O3—N2—C14—C16	70.0 (3)
C6—N3—C9—C10	−119.1 (3)	C10—N2—C14—C16	−100.1 (3)
C5—N3—C9—C8	−169.4 (3)	O3—N2—C14—C11	−171.0 (2)
C6—N3—C9—C8	2.4 (3)	C10—N2—C14—C11	19.0 (3)
C7—C8—C9—N3	19.9 (3)	N1—C11—C14—N2	−21.2 (2)
C7—C8—C9—C10	141.4 (3)	C13—C11—C14—N2	−139.5 (3)
O3—N2—C10—N1	−177.0 (2)	C12—C11—C14—N2	91.6 (3)
C14—N2—C10—N1	−7.4 (3)	N1—C11—C14—C15	−139.7 (3)
O3—N2—C10—C9	0.5 (4)	C13—C11—C14—C15	102.0 (3)
C14—N2—C10—C9	170.0 (2)	C12—C11—C14—C15	−26.8 (4)
O2—N1—C10—N2	176.7 (2)	N1—C11—C14—C16	91.4 (3)
C11—N1—C10—N2	−8.6 (3)	C13—C11—C14—C16	−26.9 (4)
O2—N1—C10—C9	−0.8 (4)	C12—C11—C14—C16	−155.7 (3)

### Hydrogen-bond geometry (Å, °)

**Table:**

*D*—H···*A*	*D*—H	H···*A*	*D*···*A*	*D*—H···*A*
C2—H2A···O1	0.96	2.47	3.043 (5)	118
C3—H3C···O1	0.96	2.43	3.025 (4)	120
C9—H11···O2	0.98	2.57	2.942 (4)	102
C16—H17C···O3^i^	0.96	2.48	3.390 (4)	157

Symmetry codes: (i) −*x*+1, *y*−1/2, −*z*+2.
